# ART attrition and risk factors among Option B+ patients in Haiti: A retrospective cohort study

**DOI:** 10.1371/journal.pone.0173123

**Published:** 2017-03-06

**Authors:** Nancy Puttkammer, Jean Wysler Domerçant, Michelle Adler, Krista Yuhas, Martine Myrtil, Paul Young, Kesner François, Reynold Grand’Pierre, David Lowrance

**Affiliations:** 1 International Training and Education Center for Health (I-TECH), Department of Global Health, University of Washington, Seattle, Washington, United States of America; 2 US Centers for Disease Control and Prevention, Port au Prince, Haiti; 3 US Centers for Disease Control and Prevention, Kampala, Uganda; 4 Center for AIDS Research, University of Washington, Seattle, Washington, United States of America; 5 Department of Global Health, University of Washington, Seattle, Washington, United States of America; 6 US Centers for Disease Control and Prevention, Atlanta, Georgia, United States of America; 7 Ministry of Public Health and Population, Government of Haiti, Port au Prince, Haiti; National Institute of Health, ITALY

## Abstract

**Objectives:**

In October 2012, the Haitian Ministry of Health endorsed the “Option B+” strategy to eliminate mother-to-child transmission of HIV and achieve HIV epidemic control. The objective of this paper is to assess and identify risk factors for attrition from the national ART program among Option B+ patients in the 12 months after ART initiation.

**Design:**

This retrospective cohort study included patients newly initiating ART from October 2012-August 2013 at 68 ART sites covering 45% of all newly enrolled ART patients in all regions of Haiti.

**Methods:**

With data from electronic medical records, we carried out descriptive analysis of sociodemographic, clinical, and pregnancy-related correlates of ART attrition, and used a modified Poisson regression approach to estimate relative risks in a multivariable model.

**Results:**

There were 2,166 Option B+ patients who initiated ART, of whom 1,023 were not retained by 12 months (47.2%). One quarter (25.3%) dropped out within 3 months of ART initiation. Protective factors included older age, more advanced HIV disease progression, and any adherence counseling prior to ART initiation, while risk factors included starting ART late in gestation, starting ART within 7 days of HIV testing, and using an atypical ART regimen.

**Discussion:**

Our study demonstrates early ART attrition among Option B+ patients and contributes evidence on the characteristics of women who are most at risk of attrition in Haiti. Our findings highlight the importance of targeted strategies to support retention among Option B+ patients.

## Introduction

In October 2012, the Haitian Ministry of Health endorsed the “Option B+” strategy to accelerate coverage of prevention of mother-to-child transmission of HIV (PMTCT) services toward the dual goals of PMTCT and achieving overall HIV epidemic control by 2030 [1,2] Under this strategy, pregnant and breastfeeding women living with HIV are routinely enrolled on lifelong HIV antiretroviral therapy (ART) regardless of immunologic or clinical status, thereby decreasing delays and barriers to ART enrollment [3,4]

HIV is among the top five causes of death and disability globally [5], and the high prevalence of HIV among adults of childbearing age results in approximately 240,000 perinatal HIV infections annually[1]. Haiti accounts for the largest number of prevalent and incident HIV infections in the Caribbean region [1], with prevalence among adult women of 2.7% [6]. In 2013, use of ARVs for PMTCT during childbirth was estimated at 87% among HIV-positive women [7]. Despite this high coverage, it is estimated that 5.8–7.7% of pregnancies still result in perinatal HIV infections, for a total of approximately 325–430 new pediatric HIV cases per year [8,9] High retention on ART under Option B+ is critical to preserving the health of pregnant and postpartum women, achieving elimination of mother-to-child HIV transmission (eMTCT), preventing transmission of HIV to sero-discordant partners, and preventing development of antiretroviral drug resistance.

Early evidence from Option B+ programs globally shows rapid expansion in PMTCT coverage [3,10,11,12,13] but with a concerning pattern of elevated attrition among those newly enrolling on ART [10,14] A retrospective cohort studies in Haiti by our research team, the parent study to the present analysis, also demonstrated this pattern[15]. Among 17,059 newly initiating ART patients, which adjusted for individual-level demographic and health characteristics, we found up to a 59% greater risk of attrition among women enrolled on ART under Option B+ compared to non-pregnant women. In the present study, we report on ART attrition outcomes after ART start following adoption of the Option B+ policy in Haiti, using the subset of the cohort with sufficient follow up time to observe 12 month outcomes. The aims of the present study are: 1) to describe ART attrition among Option B+ patients in Haiti; and 2) to examine risk factors for ART attrition among Option B+ patients, with a specific focus on the timing of HIV diagnosis, enrollment in care, and ART initiation relative to pregnancy. We also compare outcomes in Haiti with outcomes reported from other settings. The study provides an important contribution in the context of building a public health agenda, towards eMTCT and “test and treat” goals [16,17]

## Methods

### Data source

The study used longitudinal electronic medical record (EMR) data from the iSanté data system, a networked system used in more than 100 health facilities where HIV care and treatment services are provided. The iSanté system has been described elsewhere [18,19]. Facility-level iSanté data are securely replicated to a consolidated server housed within the Haitian Ministry of Health (MSPP) in Port-au-Prince on a daily basis, or as Internet connectivity permits, and the study used de-identified data from this iSanté consolidated server. Records from health facilities with out-of-date data, defined as having less than 90% of patient visit forms saved to the iSanté consolidated server within 90 days of the patient’s visit, were excluded from the analysis. Each site had a study close date based on last replication of data to the consolidated server on or before November 24, 2014. The study cohort was drawn from 68 out of 131 national ART sites, located in 9 out of 10 administrative Departments in Haiti, and covered approximately 45% of all newly-enrolled ART patients in Haiti.

### Study setting and population

Haiti’s PMTCT program has steadily evolved since the early 2000s. Starting in March 2009, for women not already enrolled on ART for their own health, the PMTCT program adopted Option B, with triple ART therapy after 28 weeks gestation through one week after cessation of breastfeeding, regardless of CD4 count. Those who met the CD4-based ART eligibility threshold of <350 cells/mm3 were advised to continue ART. Starting in June 2011, Option B was recommended after 14 weeks gestation [20]. The adoption of Option B+ in 2012 reflected MSPP’s consistent embrace of new strategies to reach national eMTCT goals. Over this period, PMTCT services extended from 113 sites in 2008 to 137 sites in 2013 [8], and ART use in PMTCT increased from 27% in 2008 to 87% in 2014 [7]. By 2014, 99% of the ARV regimens used for PMTCT were multidrug regimens suitable for life-long therapy. Early infant diagnosis services, first piloted in 2009 in Haiti, reached 3,600 HIV exposed infants in 2014, with a positivity rate of 5.3% [21].

In all of our study sites, PMTCT and ART services were located within the same health facility. In general, Option B+ patients initiated ART through the maternal health service. The national guidelines specify that transition of care to the ART service after cessation of breastfeeding, although in some facilities transition occurred earlier. Most health facilities operate a single ART pharmacy serving both PMTCT and other ART patients. The study used data on patients newly enrolling on ART from October 2012, when the Option B+ policy was considered to be operationalized in Haiti, through August 2013. Patients with at least 365 days of follow up time possible as of the health facility-specific study close date were included in the analysis. The iSanté system includes a de-duplication algorithm based on patient names and demographic details; in the case of duplicates, the record with the first ART use was retained. Patients with missing age or sex were excluded from the analysis (0.4% of records).

### Measurements

The main outcome of interest was attrition from the ART program due to death or loss-to-follow-up in the 12 months after ART initiation, under definitions used by the MSPP and the President’s Emergency Plan for AIDS Relief (PEPFAR). The MSPP definition considers patients to be cases of attrition if they made no visits for ART pick up within the 275–365 day period after ART initiation. The PEPFAR definition considers patients to be cases of attrition if: 1) after their last visit they did not have enough ARVs to last through the end of the 12^th^ month of treatment, or 2) if they died, stopped, or were lost to follow up. Both definitions count patients who drop out of care for a time after ART initiation but who return to care by 12 months as retained patients. We also considered the outcome of never returning to care after the ART start visit and not returning to care in the first 90 days post-partum. This final outcome recognizes that, among Option B+ patients, attrition as a function of time could be more closely related to timing of delivery than to timing since ART start. Known patient transfers to other health facilities, representing either patients which were coded as transfers by health workers at the index site or patients which were detected as transfers based upon the iSanté de-duplication algorithm, were excluded from the attrition analyses (n = 13, <1% patients).

The methodology for identifying Option B+ patients has been described elsewhere [15]. Briefly, the iSanté data system contains information on pregnancy and labor and delivery, but not on breastfeeding status, so patients who enrolled on ART during pregnancy or up to 12 weeks post-partum were considered as Option B+ patients. Among patients with evidence of pregnancy but with no data on last menstrual period, gestational age, or an estimated or observed delivery date, pregnancy dates were imputed, based upon the assumption that each pregnancy-related visit fell at the mid-point of a typical pregnancy of 40-weeks duration. Very few labor and delivery events are recorded within iSanté, reflecting the fact that only about one-third of deliveries take place within a health facility in Haiti [6], as well as generally low use of the iSanté EMR within labor and delivery services.

Measurement of patient demographic and clinical covariates was consistent with our earlier work [15]. Weight measures falling after the first trimester of pregnancy were not used to calculate baseline body mass index (BMI), since pregnancy-related weight gain could distort a patient’s BMI as a marker of nutritional status and health. We evaluated timing of HIV testing relative to pregnancy, timing of enrollment in HIV care and treatment relative to pregnancy, and gestational age at the time of ART initiation.

### Data analysis

We used descriptive statistical analysis to characterize the patient population, by their demographic and clinical covariates. To compare the distribution of covariates across by attrition status, we used the Chi-squared test of proportions for categorical covariates, and the Kruskal-Wallis non-parametric test for numeric covariates. We examined the timing of last visit, and analyzed interruptions in care among those who were retained at 12 months. We carried out bivariate analyses to identify whether attrition levels varied by pregnancy-related covariates (gestational age at ART initiation and timing of HIV testing relative to pregnancy). We limited these analyses to patients with more precise pregnancy start and end dates available based upon last menstrual period, gestational age, or estimated or observed delivery date. Unless otherwise specified, we used the MSPP-defined attrition outcome in our analyses.

Finally, to explore factors associated with attrition among Option B+ patients, we carried out adjusted analysis for the attrition outcome based upon the MSPP definition of retention. For all bivariate and adjusted models, we used a modified Poisson regression approach to estimate relative risks (RRs). We used generalized estimating equations (GEE) models with a log link, a Poisson family, exchangeable correlation structure, and robust variance to address correlation of data by health facility [22,23]. In the adjusted model approximately 10% of cases were complete in all covariates. We used multiple imputation by chained equations to impute missing covariate data for the GEE models. We specified 90 imputations, following the guideline that the number of imputations be equal to the proportion of incomplete cases [24]. Multiple imputation is a reasonable method when it is assumed that patterns in missingness can be explained by the observed covariates. Stata 13.1 (Stata Corp, College Station, TX) was used for all analyses.

### Ethical review

The study received scientific and ethical review and approval from the US Centers for Disease Control and Prevention and the Haiti National Committee on Bioethics, and was exempted from human subjects review by University of Washington.

## Results

There were 3,533 previously ART-naive Option B+ patients enrolled on ART from October 2012 through August 2014 in the cohort of the parent study. Of these, 2,166 women had at least 12 months of time under study following ART initiation and were included in the present analysis (Table 1). Only 968 patients (44.7%) had precise pregnancy start or end dates available, and only 850 patients (39.2%) had known dates for pregnancy and HIV testing. There were also high levels of missing data in several covariates of interest, particularly household size and presence of another known HIV+ person within the household, baseline body mass index, WHO disease stage, and hemoglobin level.

**Table 1:** Characteristics of Option B+ patients (Categorical variables).

	Option B+		Attrition^ǂ^		p-value^ǁ^
	n	%	n	%	
Total	2,166	100.0%	1,023	47.2%	
Marital status					
Married/concubinage	1,425	65.8%	683	66.8%	0.67
Widow/divorce	90	4.2%	38	3.7%	
Single	236	10.9%	107	10.5%	
Missing/Unknown	415	19.2%	195	19.1%	
Location of residence					
Different commune	647	29.9%	303	29.6%	0.70
Same commune	1,470	67.9%	694	67.8%	
Missing	49	2.3%	26	2.5%	
HIV+ household member					
No	818	37.8%	363	35.5%	<0.001
Yes	106	4.9%	35	3.4%	
Missing	1,242	57.3%	625	61.1%	
Household size					
1–3 members	698	32.2%	314	30.7%	<0.001
>3 members	226	10.4%	84	8.2%	
Missing	1,242	57.3%	625	61.1%	
WHO stage					
I	941	43.4%	468	45.7%	<0.001
II	517	23.9%	231	22.6%	
III	369	17.0%	145	14.2%	
IV	100	4.6%	44	4.3%	
Missing	239	11.0%	135	13.2%	
Period of ART start					
Oct12-Mar13	893	41.2%	401	39.2%	0.13
Apr13-Sep13	1,086	50.1%	536	52.4%	
Oct13-Mar14	187	8.6%	86	8.4%	
Starting ART regimen					
AZT-3TC-EFV	201	9.3%	82	8.0%	0.11
AZT-3TC-NVP	155	7.2%	80	7.8%	
TDF-3TC-EFV	1,752	80.9%	829	81.0%	
All Other	58	2.7%	32	3.1%	
Supportive services at ART start					
Treatment supporter named	274	12.7%	102	10.0%	<0.001
Counseling before ART start	273	12.6%	77	7.5%	<0.001
Treatment or prophylaxis for opportunistic infections at ART start					
TB prophylaxis or treatment	542	25.0%	269	26.3%	0.20
Cotrimoxizole prophylaxis	1,441	66.5%	646	63.1%	<0.01
Pregnancy					
Dates of pregnancy known*	968	44.7%	439	42.9%	0.12
Dates of HIV test and pregnancy known	850	39.2%	389	38.0%	0.27
HIV test after start of pregnancy**	687	80.8%	350	51.0%	<0.001
Registered for HIV care after preg start***	792	81.8%	398	50.3%	<0.001

^ǂ^Attrition by MSPP definition.

^ǁ^p-value for Chi-2 test statistic comparing attrition and non-attrition cases.

*Pregnancy dates were considered to be known when data were available on last menstrual period date, gestational age, estimated delivery date, or actual delivery date.

**Among cases with dates of HIV test and pregnancy known.

***Among cases with dates of pregnancy known.

AZT = Zidovudine; 3TC = Lamivudine; NVP = Nevirapine; TDF = Tenofovir; EFV = Efavirenz.

Option B+ patients had a median age of 28.1 years, and were likely to be married or in a stable partnership (65.8%), live in the same commune as the health facility (67.9%), be asymptomatic (43.4%), and be prescribed the first-line regimen of Tenofovir + Lamivudine + Efavirenz (TDF-3TC-EFV) (80.9%) (Table 1). Many were started on Cotrimoxizole prophylaxis at ART start (66.5%), but few had ART adherence counseling documented (12.6%) or a treatment supporter named at ART start (12.7%). Among those with known HIV testing dates, the median time from HIV testing to ART start was 5 days (interquartile range or IQR: 0–12 days). Among those with known pregnancy and/or HIV testing dates, HIV testing typically occurred at 15.5 weeks gestation (IQR: 7.0–23.9 weeks) while ART start typically occurred at 19.1 weeks gestation (IQR: 13.0–26.7 weeks) (Table 2). Only 18.2% of women registered for HIV care before their pregnancy (Table 1).

**Table 2:** Characteristics of Option B+ patients (Continuous variables).

	Option B+				p-value^ǁ^
	n	% missing	median	IQR	
Age at ART start (years)	2166	0%	28.1	(23.9, 33.1)	<0.001
Body mass index	285	87%	22.5	(20.5, 25.9)	0.10
Hemoglobin	1055	51%	10.7	(9.8, 11.7)	<0.01
Time: HIV test to registration (days)	1614	25%	0	(0, 1)	0.03
Time: registration to ART start (days)	2166	0%	1	(0, 62)	<0.001
Time: HIV test to ART start (days)	1614	25%	5	(0, 120)	<0.001
Gestational age at HIV test (weeks)	850	61%	15.5	(7.0, 23.9)	<0.001
Gestational age at ART start (weeks)	968	55%	19.1	(13.0, 26.7)	<0.001
	Attrition^ǂ^				p-value^ǁ^
	n	% missing	median	IQR	
Age at ART start (years)	1023	0%	27.0	(22.8, 31.9)	<0.001
Body mass index	54	95%	21.6	(20.1, 24.2)	0.10
Hemoglobin	428	58%	10.6	(9.6, 11.6)	<0.01
Time: HIV test to registration (days)	758	26%	0	(0, 1)	0.03
Time: registration to ART start (days)	1023	0%	0	(0, 18)	<0.001
Time: HIV test to ART start (days)	758	26%	2	(0, 34)	<0.001
Gestational age at HIV test (weeks)	389	62%	17.7	(11.0, 25.1)	<0.001
Gestational age at ART start (weeks)	439	57%	21.0	(14.1, 28.7)	<0.001

^ǂ^ Attrition by MSPP definition

^ǁ^ Kruskal—Wallis equality of populations rank test (with ties) comparing variable values between attrition and non-attrition cases.

**Fig 1:**
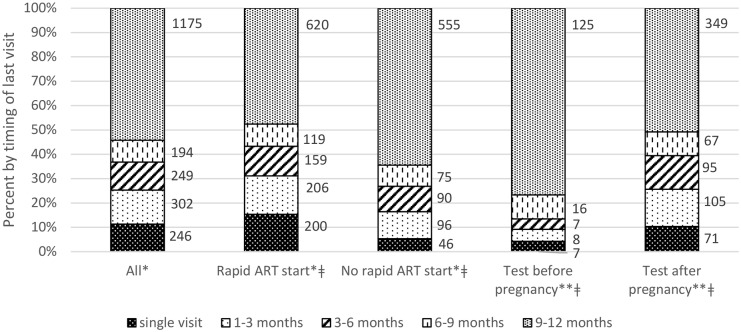
Timing of last visit in 12 months after ART start. *Among 2,166 Option B+ clients. **Among 850 Option B+ clients with known pregnancy and HIV testing dates. Rapid ART start = ART start within 7 days of registration in HIV care; No rapid ART start = ART start >7 days after registration in HIV care. ǂp<0.001 for Chi2 test of equality of proportions between Rapid ART start vs. No rapid ART start and between HIV test before vs. after start of pregnancy.

At 12 months after ART start, 1,023 (47.2%) Option B+ patients experienced attrition according to the MSPP definition, while 1,255 (57.9%) experienced attrition according to the PEPFAR definition. Many women dropped out soon after their ART start, with 246 patients (11.4%) never returning after their initial ART visit and 302 patients (13.9%) dropping out within 3 months of ART initiation (Fig 1). Even among the 1,143 patients who were retained on ART at 12 months (MSPP definition), a large proportion experienced at least one instance of being 30 or more days late for an ART pick up visit during the first year on ART (59.1%) (results not shown).

**Fig 2:**
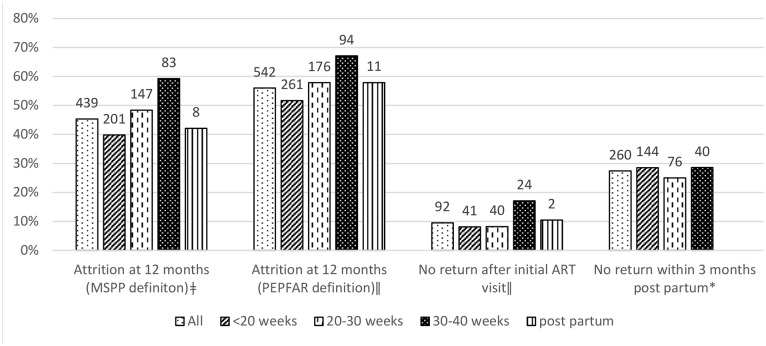
ART attrition indicators by gestational age at ART start. **Among 968 Option B+ patients with available data on pregnancy dates. ǂ p<0.001 ǁ p = 0.01 * p = 0.54 for Chi2 test of equality of proportions across gestational age groups.

Those who started ART within 7 days of enrolling in HIV care (“rapid ART start”) were at heightened risk for 12 month attrition (RR = 1.43; 95% confidence interval or CI: 1.24–1.64, p<0.001) (results not shown). They were also at risk for early attrition; specifically, 15.3% of patients with a rapid ART start never returned after their initial ART visit, while 5.3% of patients with more time before starting ART never returned (RR = 2.70; 95% CI: 2.00–3.66, p<0.001) (Fig 1). Timing of HIV testing relative to pregnancy and gestational age at ART start were also important. Among the 850 women with known pregnancy and HIV testing dates, the 687 women (80.8%) who first enrolled in HIV care after the start of pregnancy had a 2-fold risk of attrition compared to those who were diagnosed with HIV before pregnancy (RR = 2.06, 95% CI: 1.60–2.65, p<0.001) (Fig 1). Among the 968 women with known pregnancy dates, those who initiated ART in the last 10 weeks of pregnancy were at higher risk of ART attrition, compared to those who initiated in the first 20 weeks of gestation (RR = 1.52, 95% CI: 1.30–1.7; p<0.01) (Fig 2).

In the adjusted analyses, significant protective factors with respect to ART attrition were older age (RR = 0.89, 95% CI: 0.86–0.93 for each 5-year increase in age, p<0.0001), having more advanced HIV disease progression by clinical staging or baseline CD4 (RR = 0.88, 95% CI: 0.77–1.00 for stage III/IV vs. I/II, p<0.05), and having any counseling on ART adherence prior to ART initiation (RR = 0.74, 95% CI: 0.62–0.88, p<0.01) (Table 3). Significant risk factors were starting ART at 30–40 weeks gestation (RR = 1.20, 95% CI: 1.02–1.42, p = 0.03), starting ART within 7 days of HIV testing (RR = 1.19, 95% CI: 1.05–1.34, p<0.01), or having an atypical ART regimen prescribed (RR = 1.35, 95% CI: 1.08–1.69, p<0.01) (Table 3).

**Table 3:** Risk factors for ART attrition among Option B+ patients*.

	Adjusted Relative Risk	95% Confidence Interval	p-value
Marital status			
Non-married vs. married	0.97	(0.83, 1.12)	0.67
Age			
Each 5-year increase	0.89	(0.86, 0.93)	<0.0001
Household characteristics			
Live in same commune as health facility	0.94	(0.80, 1.10)	0.42
4+ members vs. 1–3 members	0.98	(0.82, 1.17)	0.81
Known HIV+ household member	0.80	(0.60, 1.06)	0.13
Timing of ART start			
Gestational age 30–40 weeks at ART start	1.20	(1.02, 1.42)	0.03
ART start within 7 days of HIV test	1.19	(1.05, 1.34)	<0.01
Period of ART initiation (reference = Oct12-Mar13)			
Apr13-Sep13	1.06	(0.95, 1.19)	0.26
Oct13-Mar14	0.97	(0.81, 1.18)	0.79
ART starting regimen (reference = TDF+3TC+EFV)			
AZT-3TC-NVP	1.06	(0.83, 1.36)	0.62
AZT-3TC-EFV	0.88	(0.72, 1.07)	0.21
All other	1.35	(1.08, 1.69)	<0.01
Clinical status at ART start			
WHO stage III/IV vs. I/II	0.88	(0.77, 1.00)	<0.05
BMI (each 1 unit increase)	0.94	(0.94, 1.01)	0.23
Moderate or severe anemia	1.08	(0.91, 1.28)	0.35
Supportive services at ART start			
Treatment buddy named	0.86	(0.70, 1.06)	0.15
Pre-ART counseling provided	0.74	(0.62, 0.88)	<0.01
Treatment or prophylaxis for opportunistic infections at ART start			
Tuberculosis-related therapy	1.03	(0.87, 1.22)	0.74
Cotrimoxazole therapy	0.94	(0.82, 1.07)	0.37

* Attrition by MSPP definition.

## Discussion

In the context of Haiti’s eMTCT agenda and the MSPP’s adoption in June 2016 of the universal “test and treat” approach to HIV care and treatment, our findings provide important evidence about sub-optimal retention on ART among women who initiate ART during or after pregnancy under Option B+. Haiti’s national PMTCT program has made important gains in recent years. Still, closing the “last mile” to achieve eMTCT will require improved retention on ART during pregnancy and post-partum periods and beyond.

Our study raises notable concerns about the high level of early ART attrition among Option B+ patients throughout Haiti. It demonstrates the profile of women at highest risk of attrition from the Option B+ program: younger women, women newly diagnosed with HIV during pregnancy or post-partum, women who initiated ART soon after diagnosis, and women who initiated ART late in pregnancy. Women who immediately move into the ART enrollment process upon learning their HIV diagnosis face the challenge of accepting their diagnosis and assimilating information about HIV treatment at the very time they are expected to start taking ART medications. Those who tested days or weeks before ART initiation would have had a greater chance to accept their diagnosis or disclose their HIV status to others, leading to lower risk of attrition. Our finding that having ART adherence counseling prior to ART initiation was protective suggests that a greater level of counseling and support at the time of enrollment is helpful; however, this finding deserves cautious interpretation. It is possible that ART adherence counseling was given in some sites, but without capturing data on this within the iSanté data system. We were unable to distinguish between the service not being offered and the data not being collected.

Our evidence on a national scale corroborates results from a study of ART retention outcomes at a single Departmental hospital in Les Cayes in Southern Haiti, during the era of the Option B approach from 2009–12 [20]. That study found elevated loss to follow up among those with HIV diagnosis during vs. before pregnancy and with ART initiation during the third trimester of pregnancy, although their results were not statistically significant in adjusted analyses. A recent mixed methods study on the PMTCT care cascade, sponsored by the MSPP and the Pan American Health Organization, investigated reasons women dropped out of PMTCT programs. The study focused on 10 health facilities in the 4 Departments of Haiti located along the border with the Dominican Republic, and was able to locate and interview 56/179 PMTCT patients (31%) who were lost to follow up from those facilities [25]. Key themes explaining loss to follow up were: lack of money to cover transport to the clinic and time away from economic activities, denial of HIV status (including a belief that a negative PCR result for the infant indicated a negative status in the mother), stigma and lack of disclosure to partners and family members, migration, ART side effects, and dissatisfaction with clinic services based upon wait time, reception, and concerns that health workers would fail to protect confidentiality.

Haiti’s experience with retention on ART following adoption of Option B+ echoes early results from other countries, as described in both quantitative and qualitative studies. In Malawi, at the start of the Option B+ program in 2011–12, 23.2% of Option B+ patients were not retained on ART after 12 months [10,26], with wide variation in outcomes across facilities [27]. In a rural area of Northern Mozambique, attrition at 12 months was reported among 64.9% of Option B+ patients [28]. Younger age [26,27,29] and rapid ART initiation following HIV diagnosis [28,30] have each been demonstrated as risk factors for attrition under Option B+ in other settings. Reasons for lost to follow up in Malawi based upon qualitative research [31,32] appear similar to reasons reported in Haiti [20,25].

In the early days of Haiti’s ART program, when only the sickest patients met ART eligibility criteria, a treatment buddy (or “accompagnateur”) model, involving directly observed therapy by community health workers, was the main model used in Haiti to reinforce ART adherence and retention in care [33]. As the ART program expanded nationally in Haiti, this model was adapted to a volunteer-based model where patients could name a friend or family member as a treatment buddy who could accompany the patient to clinic visits, collect medications on behalf of the patient, and otherwise support them. This voluntary treatment buddy model has its limitations in the context of ART use by patients who are not obviously sick, and retention support models have evolved in Haiti over time in recognition of the multi-level challenges to ART retention. Recently, the MSPP and its technical assistance partners have undertaken several promising initiatives to increase ART retention, including moves toward multi-month prescriptions and “rapid pathway” services for stable ART patients, community-based ART distribution, and active tracing of patients within 1–2 weeks of missed visits [34]. Several strategies for improving ART retention among pregnant women have shown promise in other countries, including promotion of couples testing [35,36], clinic- or community-based peer support [35,37,38,39], case management using community health workers [40], clinic level systems analysis and workflow re-design and other quality improvement approaches [35,41,42,43]. Targeted retention support initiatives for pregnant and post-partum women, especially younger women, need to be systematically tested in Haiti to reveal which innovations are most effective and cost-effective to optimize Option B+.

Strengths of our study were the large number of patients and facilities covered by the EMR data source and the ability to investigate the relationship between timing of pregnancy, HIV testing, enrollment in care, and ART start based upon patient-level EMR data. A limitation was the lack of precise pregnancy start and end dates and HIV testing dates among many patients, meaning our results on timing of ART start during pregnancy may not reflect the broader population of Option B+ patients. Another limitation was that reasons for ART discontinuation at any given health facility were only documented in 26% of non-retained patients, so transfers of care to other health facilities could have been under-reported. However, our previous analyses suggest that “silent transfer” explains <10% of cases of attrition [15]. Our findings on rapidly starting ART as a risk factor for ART attrition may reflect selection bias in that those who return to initiate ART days or weeks after HIV diagnosis may be inherently more likely to remain in care. Finally, while the study raises an interesting hypothesis about the helpful role of ART adherence counseling prior to ART start among Option B+ patients, this finding deserves cautious interpretation. Further investigation of the variation in ART enrollment processes and quality of patient education and counseling at ART initiation across health facilities under Option B+ would be helpful, and interventions to strengthen ART adherence counseling at the time of ART initiation would need to be tested via an experimental or quasi-experimental study design.

## Conclusion

Our study reports on the retention outcomes of a large cohort of newly-enrolled ART patients from 68 health facilities in Haiti following the adoption of the Option B+ policy in October 2012. Women of younger age, who tested for HIV after the start of pregnancy, who enrolled on ART late in pregnancy, who had little time between HIV diagnosis and ART initiation, and who had no pre-ART counseling were at particular risk of attrition. This highlights the importance of finding feasible strategies to support retention in care among these women, in order to fulfill the eMTCT and “test and treat” agendas in Haiti.
